# Srinivasan (1962–2021) in Bioinformatics and beyond

**DOI:** 10.1093/bioinformatics/btac054

**Published:** 2022-02-03

**Authors:** M Michael Gromiha, Christine A Orengo, Ramanathan Sowdhamini, and Janet M Thornton

**Affiliations:** Department of Biotechnology, Bhupat and Jyoti Mehta School of Biosciences, Indian Institute of Technology Madras, Chennai, Tamil Nadu 600036, India; Department of Structural and Molecular Biology, University College London, London WC1E 6BT, UK; National Centre for Biological Sciences (NCBS-TIFR), GKVK Campus, Bangalore, Karnataka 560065, India; Molecular Biophysics Unit, Indian Institute of Science, Bangalore 560012, India; Institute of Bioinformatics and Applied Biotechnology, Bangalore 560100, India; European Molecular Biology Laboratory-European Bioinformatics Institute (EMBL-EBI), Wellcome Genome Campus, Cambridge CB10 1SD, UK

Dear Editor,

Last year the Bioinformatics community lost one of its pioneers, a scientist renowned for his talent, creativity and rigour but also for his commitment to supporting his research community and particularly the young scientists he trained. He was an inspiring role model for his field and a scientist who will be remembered very fondly by his many friends in the community for his warmth, humour and kindness. For more than three decades Srinivasan developed timely and novel computational strategies for analyzing proteins and was regarded in high esteem internationally for the insights he provided and the resources he established based on the underpinning concepts. His discoveries cover many areas fundamental to structural biology and pathogen research. Although he was a computational scientist, he worked closely with experimental groups to maximize the impact of his research. He published more than 300 papers, with nearly 10 000 citations altogether.

Srinivasan joined the faculty of the Molecular Biophysics Unit, Indian Institute of Science, Bangalore in 1998, after leaving the Madras Biophysics Group (he did his Masters studies from 1982-84). He acquired his PhD degree in the Molecular Biophysics Department (the same Department where he later worked as a faculty member) within the GN Ramachandran school of peptide and peptide stereochemistry. His postdoctoral tenure was in Prof. Sir Tom Blundell’s laboratory (1991–1998), Birkbeck College, UK, with a brief stint in Prof. Mike Waterfield’s laboratory at the Ludwig Institute for Cancer Research, UK. He arrived in London as a seemingly shy young man, but it soon became clear that he was a real expert in protein structures and thought very deeply about their evolution. During these times, his research was largely focused on homology modelling (Johnson *et al.*, 1994) and the study of proteins involved in signal transduction (e.g. Srinivasan *et al.*, 1994, 1996). After coming back to MBU, he headed the ‘Proteins: structure, function and evolutionary’ group. He made major contributions to the understanding the structure and functions of proteins, particularly on protein kinases in a wide range of model organisms (e.g. Krupa and Srinivasan, 2002; Krupa *et al.*, 2004a, b). Specifically, his lab was focused on computational genomics, bioinformatics and structural biology, particularly involving the relationships between protein structure, function and interactions, including protein–protein interactions, cellular signal transduction and biological pathways.

Srinivasan’s interest in protein families and protein evolution drove research into strategies for improving multiple alignments of relatives and for better characterizing phylogenetic relationships (which resulted in many useful resources like SUPFAM, MulPSSM, PALI and DoSA). It also drove the design of methods to detect extremely remote homologues, which have been valuable for extending structural and functional annotation of genomes. Srinivasan’s group also showed that sequence-based connections of distantly related proteins can be enabled through the design of artificial sequences (Mudgal *et al.*, 2014). This work was highly innovative and can help to bring valuable annotations for pathogen proteins, which are typically difficult to characterize by more conventional, less-sensitive strategies. His strategies allowed a much deeper characterization of fold space to inform protein engineering.

Srinivasan is also highly renowned for his analyses of how changes in the protein structure and sequence impact function. Some protein families, like the kinases, were a major focus of his research and gave him much international acclaim. He studied kinases for >20 years and contributed numerous insights important for understanding their mechanisms and for enabling drug design. For example, structural fluctuations, classifications based on key functional site properties (Kalaivani *et al.*, 2018), mechanisms of stabilization of their key functional sites through specific residue interactions. He also characterized the ways in which domain partnerships modify structure (Vishwanath *et al.*, 2018), kinase functionality and characterized how splicing extends the kinase functional repertoire. This large family is implicated in many human diseases, including cancer, and these discoveries have informed drug design.

However, the biological role of proteins is determined by their interactions and Srinivasan applied his precise analytical skills in this arena, too, revealing key insights into the properties of the interfaces involved in assembling protein complexes. He produced a substantial body of very rigorous studies, including analyses of the characteristics of transient complexes and the effect of protein associations on global structural dynamics. He robustly captured this knowledge in the PIC protein interactions calculator (Tina *et al.*, 2007), a valuable tool that is freely available to biologists and very popular among researchers to obtain structural data on various non-covalent interactions within a protein or between proteins in a complex. An important application of these methods was the characterization of interactions between viral proteins and their host proteins, which provided key data for understanding pathogenicity and enabling drug design. For example, Srinivasan performed various studies characterizing toxin–antitoxin systems (Tandon *et al.*, 2019), protein interactions between human erythrocytes and *Plasmodium falciparum* and *Helicobacter* *pylori* and human.

**Figure:**
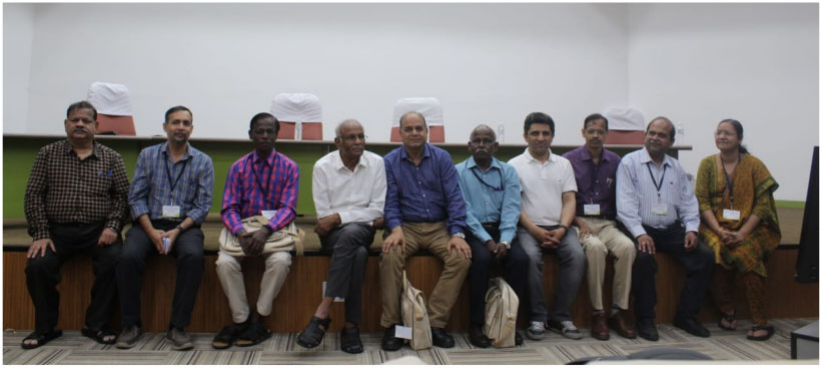
Photo taken at the fifth IIT Madras-Tokyo Tech joint symposium on ‘Current Trends in Bioinformatics: Big Data Analysis, Machine Learning and Drug Design’ with leading Bioinformatics scientists in India (March 2020). From left to right D. Velmurugan, N. Manoj, S. Selvaraj, P.K. Ponnuswamy, G.P.S. Raghava, K. Veluraja, Shandar Ahmad, M. Michael Gromiha, N. Srinivasan, R. Sowdhamini

In collaboration with multiple laboratories, Srini’s group studied fascinating biological systems, including protein assemblies of ribosomes and spliceosomes (Bhat *et al.*, 2015; Pudi *et al.*, 2003; Yazhini *et al.*, 2022) and developed powerful computational tools for studying structures of large assemblies derived from cryo-electron microscopy (Joseph *et al.*, 2016; Rakesh *et al.*, 2016). As is true of several structural bioinformaticians, his group relied on publicly available structural data and were concerned with the quality of protein–ligand data, deposited through X-ray or cryo-EM studies (Chakraborti *et al.*, 2021). He was always excited to discuss the Ramachandran map. Last year, he attended the fifth IIT Madras—Tokyo Tech joint symposium on Bioinformatics and his lecture on the Ramachandran map was fascinating. Using modern computational tools and along with late Prof. C. Ramakrishnan (his PhD mentor and the student behind the original Ramachandran map) and one of his students Ashraya Ravikumar, he re-examined the classical and renowned Ramachandran map. They clearly demonstrated that it is possible to consider deviations in the ‘allowed’ regions within this map by considering slight deviations in internal parameters from ideal values of the peptide bond (Ravikumar *et al.*, 2019).

Srinivasan’s group also participated in several consortia such as the Open Source Drug Discovery program [with the groups of Prof. Tom Blundell (University of Cambridge, UK), Nagasuma Chandra (Indian Institute of Science, India) and Sowdhamini (National Centre for Biological Sciences, India)], the UKIERI study of protein assemblies [with the groups of Profs. Jim Warwicker (University of Manchester, UK), Pinak Chakrabarti (Bose Institute, India), Nagasuma Chandra and Sowdhamini)] and collaborations such as the Indo-French CEFIPRA project on protein alphabets [with Dr. Alexandre de Brevern (INSERM Paris, France) and Dr. Bernard Offmann (University of Nantes, France)], and the Centre for Excellence on protein-protein interactions [with Profs. Sowdhamini and Satyajit Mayor (National Centre for Biological Sciences, India) and Nagasuma Chandra (Indian Institute of Science, India)] and toxin-antitoxin systems [with Prof. Raghavan Varadarajan (Indian Institute of Science, India)].

Throughout his career Srinivasan applied his knowledge, data and computational tools to characterize the protein structures, functions and virus–host interactions of multiple pathogenic bacteria affecting human health, including mycobacterial pathogens (e.g. Mtb), malaria, *H. Pylori*, Dengue and several gut pathogens. Understanding the critical residues in the protein interface is essential for drug design to reduce infection and pathogenicity. For many years he collaborated with the group of Professor Tom Blundell in Cambridge, UK. As well as detailed analyses, he established the SInCRe structural interactome resource for Mtb in 2015 (Metri *et al.*, 2015), which contributed to studies on the repurposing of drugs for this pathogen. His tools have been applied in a number of medical contexts with promising clinical results. His group also applied docking tools to FDA-approved drugs to SARS-CoV2 (Chakraborti *et al.*, 2020) and his most recent work on inhibitors for the main protease of SARS-CoV2 led to compounds already in clinical trials.

Prof. Srinivasan made significant contributions to Bioinformatics and his whole-hearted involvement in scientific activities will not be forgotten. As a scientist, Srinivasan was very highly focused and meticulous. He always set high standards—whether in creating high-quality datasets or in his interpretations of data or in responding to reviewers’ comments. As well as being a multitalented and well-known researcher, Srinivasan actively participated in many university and external committees, commented on PhD theses, and delivered popular and invited lectures in most of the leading conferences in India. He was an elected fellow in all the three major academies in India (Indian National Science Academy, New Delhi, National Academy of Sciences, Allahabad and Indian Academy of Sciences, Bangalore). He also received the most prestigious awards in India including Shanti Swarup Bhatnagar Prize for Science and Technology from Council of Scientific and Industrial Research, National Bioscience Award from the Department of Biotechnology and J.C. Bose National Fellowship from the Department of Science and Technology, Government of India.

Srinivasan had the special ability to cordially relate with others he respected and had a very positive attitude towards the work of his fellow researchers. He was a faithful chairperson of the Department (serving between 2018 and 2020) and always supported and wished his younger colleagues to do well. He remained active even when he was critically ill. During this time he still managed to publish around 10 papers, enable six of his lab colleagues to reach higher positions and also attended to multiple student-thesis-related matters. He was very enthusiastic about his research on protein structures and his ability to explain major concepts in a simple accessible manner was extremely impressive. He had a passion for naming his students with ‘amino acids’, each with a background story and spent considerable time with his students in the midst of his busy schedule. He always encouraged young researchers and provided valuable advice for their research.

He also had an uncanny enthusiasm and ability to make sure that the people around him felt included and important—he would not hesitate to talk to prospective students, spend a long time on discussions with visitors and provide his undivided attention on work discussions with his students. Many of his students travelled widely and benefitted laboratories and science throughout the world. His students made a large impact wherever they went because their knowledge was always deep and impressive and they showed great enthusiasm for their work, mirroring their mentor. They often liked to discuss their work in detail and place it in the wider context of global knowledge. Although based in India for most of his career, Srinivasan travelled widely and has had an impact on many scientists involved in protein structure analysis. He was also a great host for visitors, ensuring their well-being and spending time discussing their work and ideas.

Srinivasan had been a very special and unusual personality—with unlimited affection and love for the people around him. He was able to sense people in trouble and would often go out of his way to help them. His smiling face is not forgettable at any time and evidenced a deeply contented person, very proud of his family, his students and his science and always happy to discuss anything to do with proteins. His positivity and passion for science were infectious. His too-early passing is a great loss to science and to everyone who knew him.

*Financial Support*: none declared.

*Conflict of Interest*: The authors declare that there are no conflicts of interest. 
